# Development and validation of the psychological stability scale for special operations personnel in China

**DOI:** 10.3389/fpsyg.2026.1731159

**Published:** 2026-02-26

**Authors:** Lingzhi Wang, Chunxiao Ren, Zhengzhi Feng, Xiaoping Du, Kebei Chen, Xiaodi Han

**Affiliations:** 1 Experimental Research Center of Medical and Psychological Science, School of Psychology, Army Medical University, Chongqing, China; 2 Department of Medical Psychology, First Affiliated Hospital, Army Medical University, Chongqing, China; 3 School of Medicine, Chongqing University, Chongqing, China; 4 School of Nursing, Army Medical University, Chongqing, China

**Keywords:** grounded theory, psychological stability scale, scale development, special operations personnel, validation

## Abstract

**Introduction:**

Special operations personnel are consistently exposed to high-stress, high-risk, and hazardous occupational environments due to the unique operational demands. To effectively cope with these stresses and ensure successful task execution, high levels of psychological stability are required of them. However, specific assessment tools are lacking to evaluate the psychological stability of special operations personnel. Based on grounded theory, this study proposed a theoretical model of psychological stability among special operations personnel and developed a corresponding measurement scale.

**Methods:**

A mixed-methods approach integrating both qualitative and quantitative research was adopted across three studies. Study 1 included in-depth interviews conducted with 30 special operations personnel. Grounded theory was applied to analyze the dimensions of psychological stability, and the Delphi consensus was used to determine the specific connotation of each dimension of psychological stability. Study 2 focused on developing and refining the scale through content validity analysis, item analysis, item-total correlation analysis and exploratory factor analysis. Study 3 validated the scale using confirmatory factor analysis and conducted reliability and validity testing.

**Results:**

Study 1 identified seven dimensions of psychological stability, including self-confidence, conscientiousness, equanimity, rationality, disturbance tolerance, stress tolerance, and frustration tolerance. In Study 2, a 29-item psychological stability scale (PSS) with five dimensions was developed, including self-confidence, conscientiousness, equanimity and rationality, disturbance tolerance, stress and frustration tolerance. Study 3 demonstrated that the scale exhibited strong reliability and validity across different samples.

**Discussion:**

This study innovatively revealed the dimensions of psychological stability in special operations personnel and developed a valid and reliable measurement tool.

## Introduction

Special operations personnel refer to individuals directly engaged in special operations, and are frequently exposed to high-pressure, high-risk, and hazardous occupational environments, with numerous safety risks (Wu et al., 2024). In fact, approximately 70 to 80% of accidents triggered by human error are caused by psychological factors (Mahaju et al., 2023). Stability is a core physiological and psychological indicator of occupational safety and adaptability for special operations personnel (Wu et al., 2024). Previous studies have found that psychological stability is closely related to individual physical and mental health as well as organizational effectiveness (Travis et al., 2018). It has been identified as one of the most important characteristics for managers to make effective decisions in extreme situations (Nazarov and Mitina, 2023). When individuals encounter life-threatening events, psychological stability serves as a protective factor that enhances the ability to adapt to environmental challenges and mitigates the adverse effects of stress (Spytska, 2024). Psychological stability of military personnel is also considered a key factor in national security (Abduraupovich, 2025) reflecting their ability to perform tasks under difficult conditions such as harsh combat environment, adverse factor pressure, and psychological stress (Hunchenko et al., 2025). Meanwhile, psychological stability is an important contributor to success of professional athletes in competition and their continuous improvement (Rakhimova, 2025). Individuals who maintain high psychological stability can ensure the successful execution of activities and the appropriate responses to stimuli in an isolated and closed environment (Kuznetsova et al., 2016), and can objectively assess situations and take reasonable actions to solve problems (Kuznetsova et al., 2018). Therefore, investigating the psychological stability of special operations personnel has important theoretical and practical implications for promoting their physical and mental health and ensuring occupational safety.

The earliest description of psychological stability appeared in H. Eysenck’s work on neuroticism, in which he proposed that psychological stability reflects the excitation/inhibition process of the nervous system, and that imbalance in this process is the main factor leading to the development of psychosomatic diseases (Belasheva and Petrova, 2016). Subsequently, scholars have proposed various definitions of psychological stability. Palamarchuk and Haba (2024) posited that psychological stability is a complex and multi-factor personality trait, encompassing emotional, volitional, motivational and cognitive components. According to Kuznetsova et al. (2018), psychological stability refers to the ability to successfully carry out professional activities under difficult and extreme circumstances as under normal ones. Sayfullaev (2023) emphasized its role in withstanding stress and extreme situations. Dahi and Kareem (2022) defined it as the consistency of emotional states and the absence of frequent emotional fluctuations. Hamid et al. (2024) linked psychological stability to personal value, peace of mind, and self-confidence. Rahmadi et al. (2025) included psychological resilience, subjective well-being, and adaptability as the dimensions of psychological stability. Rakhimova (2025) argued that psychological stability encompasses emotion management in a competitive context, appropriate behavior in stressful situations, sustained self-confidence, and maintenance of a stable psychological state. However, to date, there is still no widely consensus definition of psychological stability (Al-Rousan et al., 2023). Therefore, it is imperative to develop a conceptual and dimensional structure of psychological stability (Spytska, 2024).

Currently, three approaches are employed to assess psychological stability. The first is the indirect assessment using scales related to psychological stability, such as the Big Five Personality Inventory (Scharbert et al., 2024), the Brief Resilience Scale (Rustamov et al., 2023), the Depression, Anxiety and Stress Scale-21 (AbuAlula et al., 2023). The second combines multiple scales, such as combining the Emotional Intelligence Questionnaire with the Sport Anxiety Scale (Palekar et al., 2023), the Patient Health Questionnaire-9 (PHQ-9) with the Generalized Anxiety Disorder-7 (GAD-7) (Ji et al., 2023), or the Tolerance of Uncertainty Scale with the Psychological Well-being Scale (Trofimov and Zabolotna, 2023). The third involves evaluating psychological stability using subscales or dimensions from certain scales, such as using a subscale of the Stanford Integrated Psychosocial Assessment for Transplantation, which is applicable to assessing psychological stability (Takano et al., 2023), or using a revised Hospitalization Coping Scale with a newly added psychological stability dimension (Lyu et al., 2017). While these methods can be adopted to assess psychological stability, they still have some limitations. First, they only assess psychological stability indirectly. Second, they only measure some specific components of psychological stability and fail to reflect its overall structure. Finally, combining multiple scales results in a large number of items, which is time-consuming. Thus, it is essential to develop a psychological stability scale (PSS) with sound reliability and validity by following the scientific protocols.

At present, however, research on the concept, dimensions and evaluation of psychological stability among special operations personnel remains limited. Therefore, this study focuses on special operations personnel—a group for whom safety is paramount—to explore the dimensions of their psychological stability and to develop an evaluation tool for it, to provide a basis for the occupational selection of special operations personnel and for accident prevention. This research followed the process below: (1) Developing theoretical dimensions of psychological stability for Chinese special operations personnel via grounded theory-based qualitative research; (2) Constructing an item pool based on qualitative research outcomes and existing scales, followed by developing the PSS through content validity analysis, item analysis, item-total correlation analysis, and exploratory factor analysis (EFA); (3) Conducting cross-sample validation of the PSS via confirmatory factor analysis (CFA) and reliability and validity tests.

## Study 1: constructing dimensions of the psychological stability for special operations personnel using grounded theory

To gather perspectives on psychological stability among special operations personnel, this study conducted in-depth interviews with them. The collected textual data were then systematically coded, and the structural dimensions were analyzed using grounded theory.

### Methods

#### Formulation of the interview guide

In-depth interviews were employed in this study. The research team, comprising two psychology professors, two psychology master’s students, one management doctoral student, and one special operations service member, developed a preliminary interview outline through group discussions. Three pilot interviews were then conducted to refine the outline based on the feedback from participants, resulting in the final formal interview guide. Key interview questions included: “What do you think psychological stability refers to?” “What do you think psychological stability consists of?” “Do you consider yourself a person with high or low psychological stability?” “What events in your life affect your psychological stability?” and “What events at work affect your psychological stability?” These questions were aimed at comprehensively obtaining special operations personnel’s in-depth interpretation of psychological stability.

#### Participants

This study carried out field investigations following the principle of theoretical sampling. The inclusion criterion for participants was current employment as special operations personnel. The exclusion criteria were: (1) presence of major physical disabilities, and (2) presence of mental diseases and psychological disorders. Finally, 30 participants were recruited, including 25 males and 5 females, with 19 married and 11 unmarried individuals. Their ages ranged from 23 to 39 years (*M* = 29.37, SD = 3.86), and their length of service ranged from 2 to 21 years (*M* = 10.23, SD = 4.13).

#### The process of in-depth interview

Before conducting the formal interviews, the research team formed an interview group consisting of one management doctoral student and one psychology master’s student, both of whom received systematic training. Two weeks before the interviews, the interviewers shared the interview outline with the head of the special operations personnel, who briefed the participants on the study’s details. The location of the interview was chosen based on the principle of convenience and safety of the participants and freedom from interruptions during the interview. The interview time was scheduled at the participants’ convenience. Formal interviews were conducted in a one-to-one and face-to-face format, with audio recording after consent was obtained. Each interview was paused when no new concepts emerged. The research team transcribed the recordings immediately after each interview; the transcripts were then coded. Whether to continue the interview was based on the quality of the coding results. This process was repeated until theoretical saturation was achieved. The average duration of the interviews was 46 min (range: 40 min 43 s to 1 h 5 min 46 s). Ultimately, approximately 320,000 words of interview transcripts were collected.

#### Grounded theory

In qualitative research, grounded theory is considered a relatively scientific methodology. Its application can effectively solve problems including insufficient standardization of research methods, challenges in tracing and verifying research processes, and limited persuasiveness of conclusions. Classical grounded theory, one of the three major paradigms of grounded theory, places emphasis on avoiding preexisting assumptions throughout the research process and enabling endogenous theories to emerge naturally from empirical contexts, which is considered an approach embodying the most empirical spirit (Xudong, 2016). Given the inconsistent understanding of psychological stability in existing literature, this study adopted the classical grounded theory to explore the structural dimensions among special operations personnel, ensuring objectivity and scientific rigor.

#### Data analysis

The qualitative analysis software NVivo 11.0 was used to code (open, axial, and selective coding) and statistically analyze the collected interview data in this study (Liu et al., 2025).

### Results

#### Open coding

Open coding demands a thorough comprehension of the discourse system within textual materials, decomposing the text into independent nodes with distinct meanings, to realize the deconstruction of texts into specific and meaningful units (Chen, 1999). In this study, 96 codes were identified by open coding, covering 712 reference points (see Table 1).

**Table 1:** Distribution of coding nodes for psychological stability.

Codes	Open coding	Reference points (n)	Axial coding	Selective coding
1	Self-confidence	15	Self-confidence	Self-confidence
2	Champion is mine for the taking	1		
3	Self-doubt	1	Self-skepticism	
4	Self-distrust	1		
5	Responsibility	2	Responsibility	Conscientiousness
6	Be responsible for his work	1		
7	Willing to bear the adverse consequences	1		
8	Sacrifice oneself for others	1		
9	High standards for myself	4	Self-discipline	
10	Obey rules	1		
11	Caution and prudence	2	Secure and trustworthy	
12	Give others a sense of security	2		
13	Be trustworthy	4		
14	Mental equanimity	10	Mental equanimity	Equanimity
15	Inner peace	3		
16	Imperturbable	5		
17	Nervousness and anxiety	112		
18	Impatient and irritable	45		
19	Fear and dread	28		
20	Overwhelming excitement	8		
21	Stay calm in any situation	11		
22	Being calm and composed	1		
23	Being mature and steady	24		
24	Complaining bitterly	17		
25	Be emotionally stable	53	Emotional stability	
26	Fluctuating moods	14		
27	Controlling emotions	37		
28	Emotional adjustment	10		
29	Emotional management	4		
30	Emotional expression	1		
31	Outburst of temper	4		
32	Emotional response	2		
33	Keep a relaxed mindset	6	Relaxed mindset	
34	Take gains and losses in stride	2		
35	Neither conceited nor rash	1		
36	Consider comprehensively	11	Comprehensive consideration	Rationality
37	Careless consideration	4		
38	Far-sighted	1		
39	Put yourself in others’ shoes	2		
40	Rigorous and meticulous mind	1		
41	Have a clear plan in mind	1		
42	Objective Analysis	2	Objective Analysis	
43	Rational Analysis	9		
44	Careful and serious analysis	1		
45	Insight into the Essence	3		
46	Pinpoint the crux of the problem	5		
47	Rational judgment	2	Reasonable judgment	
48	Making calm judgments	2		
49	Making clear judgments	1		
50	Making correct judgments	1		
51	Making wrong judgments	3		
52	Making wise decisions	1	Wise decision-making	
53	Making the right decisions	2		
54	Devising reasonable solutions to problems	18		
55	Making decisions quickly	1		
56	Hesitant about making decisions	1		
57	Making the opposite decisions	1		
58	Improper decision making	5		
59	Making the wrong decisions	1		
60	Rational Disposal	3	Rational handling	
61	Handled appropriately	6		
62	Decisive in handling problems	6		
63	Proposing perfect measures	28		
64	Methodical and orderly	12		
65	Adopting different disposal methods	4		
66	Improper disposal	3		
67	Resistance to external interference	2	Resistance to interference	Disturbance tolerance
68	Mental clutter	1		
69	Interruptions can easily lead to mistakes	1		
70	The power of insensitivity	2		
71	Focusing attention	19	Concentration	
72	Focusing energy	3		
73	Maintaining concentration	11		
74	Easily distracted	2		
75	Overthinking trivial matters	1		
76	Be prone to external influences	7	Susceptible to influence	
77	Be easily affected by trivial matters	1		
78	Ability to work under pressure	35	Stress resistance	Stress tolerance
79	Bearing capacity	15		
80	Resistance to pressure	1		
81	Consistent handling capability	1		
82	Chain reaction	1	Negative Generalization	
83	Always expecting the worst outcome	1		
84	Everything in my body feels abnormal	1		
85	Defenses collapse	2		
86	Ability to withstand adversity	12	Frustration tolerance	Frustration tolerance
87	Frustration of desires	1		
88	Overcome difficulties	1	Overcoming obstacles	
89	Defeat difficulties	1		
90	Embrace the result	1	Embrace reality proactively	
91	Face up to reality	4		
92	Accept critical feedback	1		
93	Get stuck on difficulties	4		
94	Quick recovery	2	Resilience and Development	
95	Restoring balance	1		
96	Continuously growing	2		
	Total	712		

#### Axial and selective coding

Since the 96 codes represented a collection of different meaning units, they needed to be systematically analyzed. The 96 codes were integrated into 22 axial codes through similarity comparison, difference comparison, horizontal comparison, and vertical comparison. Subsequently, selective coding integrated and refined the axial codes at a higher level of abstraction to generate core categories related to the topic. Centering on the theme “conceptual dimensions of psychological stability,” 7 selective codes were established based on 22 axial codes, including self-confidence, conscientiousness, equanimity, rationality, stress tolerance, disturbance tolerance, and frustration tolerance (Table 1). Focusing on this research topic facilitated the construction and exploration of connections between categories, thereby deepening the understanding of psychological stability. Table 2 presents the typical codes of interview data corresponding to these dimensions.

**Table 2:** Interview examples for each dimension of psychological stability.

Dimension	Source semantic examples
Self-confidence	After my competitors finished their assignments, I saw their work was far from matching my level, and this made me feel particularly psychologically stable, and I knew this championship was definitely mine for the taking. (*S27*)
	The self-doubt and self-denial I felt at that time meant I was psychologically unstable. (*S28*)
	If I clearly know that I cannot do a certain task well, I think that will affect my psychological stability. (*S19*)
Conscientiousness	If I’m psychologically unstable, my sense of responsibility at work will diminish, and I’ll adopt a half-hearted attitude. (*S07*)
	First, he (the leader) is accountable for his work and responsible to his subordinates, so his psychological stability is better than that of his subordinates. (*S18*)
	Before carrying out high-risk missions, I must consider the consequences—even if they go badly, I’ll bear the responsibility. (*S24*)
Equanimity	Being unable to stabilize one’s mental state is essentially being unable to stabilize one’s emotions. (*S01*)
	Psychologically stable individuals can maintain a normal state of mind. (*S06*)
	If you are constantly anxious, your psychological stability may be relatively poor. (*S02*)
Rationality	Individual who cannot correctly and rationally face this matter and thus make a wrong decision. (*S14*)
	I can stabilize my mental, conduct rational analysis, and take rational actions. (*S26*)
	I feel that psychological stability is needed for making a calm and rational judgment about a matter. (*S05*)
Disturbance tolerance	The first aspect of psychological stability is resistance to interference. For example, when I am concentrating on work, the noise around me cannot distract me. (*S03*)
	When engaged in a highly challenging job, I believe one needs to have a very stable mental state and focus on doing it. (*S05*)
	If we possess high psychological stability and eliminate internal distractions, we can reduce work errors. (*S26*)
Stress tolerance	A person’s ability to handle stress is directly related to their psychological stability. (*S01*)
	Even when work pressure is high, I can still maintain my usual work performance. (*S03*)
	Psychological stability refers to the ability to maintain your usual state to cope when encountering something. (*S25*)
Frustration tolerance	When encountering difficulties at work, one should overcome them rather than be defeated by them. (*S02*)
	When facing major setbacks, if you get through them, your mental state will be more stable. (*S08*)
	I made a mistake in my last competition, but it will not affect my next one. I believe this is the result of psychological stability. (*S28*)

*S01* stands for the first respondent, and so forth.

#### Delphi consensus

Drawing on the data from interviews and the results of coding, this study defined the connotations of the seven dimensions of psychological stability. Two rounds of expert consultation on their importance were then conducted. A total of 30 experts participated in the first round of Delphi consensus, with an average age of 49.90 (SD = 6.87), and the average length of service was 22.30 (SD = 8.24). The first round included 18 experts in psychology and 12 experts in special operations. Furthermore, the second round invited 17 experts to participate, including 11 experts in psychology and 6 experts in special operations. The average age of experts was 48.35 (SD = 7.47), and the average length of service was 25.47 (SD = 7.86). The mean expert authority coefficient was 0.87 in the first consultation round and 0.86 in the second, while the response rate was 100% in both rounds. Based on the connotation of each dimension of psychological stability, experts evaluated the importance of each dimension via a 5-point Likert scale (from 1 = “not important” to 5 = “very important”) and provided suggestions for revising their names and definitions. The results of expert consultation are shown in Tables 3, 4, and the coefficient of variation of all dimensions was less than 0.25, indicating that all the seven dimensions of psychological stability constructed in this study were important.

**Table 3:** Consultation results on the importance of psychological stability dimensions.

Dimensions	The first round (*N* = 30)				Dimensions	The second round (*N* = 17)			
	*SD*	*M*	*CV*	*FSF*		*SD*	*M*	*CV*	*FSF*
Self-confidence	0.254	4.933	0.051	0.933	Self-confidence	0.332	4.882	0.068	0.882
Conscientiousness	0.490	4.633	0.106	0.633	Conscientiousness	0.507	4.412	0.115	0.412
Emotional regulation	0.305	4.900	0.062	0.900	Equanimity	0.437	4.765	0.092	0.765
Decision making	0.718	4.633	0.155	0.733	Rationality	0.562	4.765	0.118	0.824
Disturbance tolerance	0.504	4.767	0.106	0.800	Disturbance tolerance	0.332	4.882	0.068	0.882
Stress tolerance	0.504	4.767	0.106	0.800	Stress tolerance	0.393	4.824	0.082	0.824
Frustration tolerance	0.379	4.833	0.078	0.833	Frustration tolerance	0.393	4.824	0.082	0.824

SD stands for standard deviation, M for mean, CV for coefficient of variation, and FSF for full-score frequency.

**Table 4:** Kendall’s coefficient of concordance for the Delphi consensus.

Dimensions importance	*W*	*χ^2^*	*df*	*P*
The importance of dimensions in the first round	0.099	17.779	6	0.0068
The importance of dimensions in the second round	0.266	27.171	6	0.0001

W stands for Kendall’s coefficient of concordance, *c*^2^ for Chi-square value, and df for degrees of freedom.

#### Theoretical model construction

In this study, using grounded theory methodology as the primary method, supplemented by the Delphi method, seven dimensions of psychological stability for special operations personnel were progressively constructed, namely self-confidence, conscientiousness, equanimity, rationality, disturbance tolerance, stress tolerance, and frustration tolerance. The connotations of these seven dimensions were defined as follows: (1) self-confidence refers to the trait of individuals who believe in and affirm their own abilities. (2) Conscientiousness refers to the trait of individuals who consciously fulfill their obligations, actively take responsibility for the consequences of their own behavior, and have the courage to undertake the mission. (3) Equanimity refers to the trait of individuals who can maintain emotional stability, peace and calm through emotional regulation strategies. (4) Rationality refers to the trait of individuals who can make wise decisions through comprehensive consideration, objective analysis, and reasonable judgment. (5) Disturbance tolerance refers to the ability of individuals to effectively regulate attention stability and flexibility in the face of external noise, temptation and other disturbances in the process of achieving goals. (6) Stress tolerance refers to the ability of individuals to maintain the normal functioning of their psychological and physiological functions when they believe that their own abilities may be difficult to meet external demands. (7) Frustration tolerance refers to the ability of individuals to quickly restore their psychological balance and actively respond to the realistic failure when their goals are blocked or expectations fall short.

According to Allostasis, when individuals face changes in the internal and external environment, they adjust their physiology, psychology and behavior based on the original homeostasis, to establish and maintain a new homeostatic balance adaptive to the environment (Gao and Yu, 2020). Therefore, we believe that self-confidence, conscientiousness, equanimity, and rationality may be considered as the basis of internal homeostasis. Stress tolerance, disturbance tolerance, and frustration tolerance may be the external adaptive system of psychological stability. The seven factors cooperate with each other to enable individuals to effectively buffer the impact of stressors or to quickly restore psychological balance and adapt to new environments after the impact. Thus, psychological stability is defined as the psychological qualities (self-confidence, conscientiousness, equanimity, rationality) and psychological capabilities (stress tolerance, disturbance tolerance, frustration tolerance) that enable an individual to regulate and control their mental processes effectively, thereby maintaining normal functional performance and psychological well-being under stressful or challenging conditions.

#### Theoretical saturation testing

After completing the coding process, we conducted supplementary interviews with two participants. The interview data were then coded and analyzed, and no fresh conceptual categories emerged. Thus, it can be confirmed that the dimensions of psychological stability have achieved theoretical saturation.

## Study 2: development of the PSS

The aim of this study was to quantitatively validate the dimensions of psychological stability initially established via grounded theory. Therefore, the initial version of the PSS that contained 39 items was determined through content analysis combined with research team discussion, and the scale administration was conducted. Through item analysis, item-total correlation analysis, and EFA, the scale was optimized to a final 29-item version.

### Methods

#### Items determination

We developed the item pool for the PSS based on the results of Study 1, referring to the existing scales, such as the Big Five Personality Scale and the Emotion Regulation Questionnaire. There were 71 items in the initial item pool. Ten experts (4 in psychology, 6 in special operations) were invited to evaluate item-dimension relevance. Their mean age was 44.60 (SD = 7.68) years, with a mean work experience of 17.70 (SD = 9.08) years. The mean authority coefficient was 0.88, and response rate of experts was 100%. Experts rated the relevance of each item on a 4-point Likert scale (1 = “not relevant” to 4 = “very relevant”). We conducted content validity analysis on the expert ratings, excluding one item that was not up to standard (I-CVI = 0.70, Pc = 0.117, K* = 0.660). The remaining 70 items met the standard. However, considering that too many test items will increase the burden of the participants and reduce their cooperation, 39 items out of 70 items were selected to form the initial version of the PSS through group discussion (see Table 5). There were 5 items in the dimension of self-confidence, 5 items in the dimension of conscientiousness, 5 items in the dimension of equanimity, 6 items in the dimension of rationality, 6 items in the dimension of disturbance tolerance, 6 items in the dimension of stress tolerance, and 6 items in the dimension of frustration tolerance.

**Table 5:** Content validity analysis of preliminary items.

Preliminary version item number	*I-CVI*	*Pc*	K^*^	Evaluation of K*
Self-confidence-1	1.00	0.001	1.000	Excellent
Self-confidence-2	0.90	0.010	0.899	Excellent
Self-confidence-3	0.90	0.010	0.899	Excellent
Self-confidence-4	0.90	0.010	0.899	Excellent
Self-confidence-5	1.00	0.001	1.000	Excellent
Conscientiousness-1	1.00	0.001	1.000	Excellent
Conscientiousness-2	1.00	0.001	1.000	Excellent
Conscientiousness-3	1.00	0.001	1.000	Excellent
Conscientiousness-4	1.00	0.001	1.000	Excellent
Conscientiousness-5	1.00	0.001	1.000	Excellent
Equanimity-1	0.90	0.010	0.899	Excellent
Equanimity-2	1.00	0.001	1.000	Excellent
Equanimity-3	1.00	0.001	1.000	Excellent
Equanimity-4	1.00	0.001	1.000	Excellent
Equanimity-5	1.00	0.001	1.000	Excellent
Rationality-1	0.90	0.010	0.899	Excellent
Rationality-2	1.00	0.001	1.000	Excellent
Rationality-3	1.00	0.001	1.000	Excellent
Rationality-4	1.00	0.001	1.000	Excellent
Rationality-5	0.80	0.044	0.791	Excellent
Rationality-6	0.90	0.010	0.899	Excellent
Disturbance tolerance-1	1.00	0.001	1.000	Excellent
Disturbance tolerance-2	1.00	0.001	1.000	Excellent
Disturbance tolerance-3	1.00	0.001	1.000	Excellent
Disturbance tolerance-4	0.90	0.010	0.899	Excellent
Disturbance tolerance-5	1.00	0.001	1.000	Excellent
Disturbance tolerance-6	0.90	0.010	0.899	Excellent
Stress tolerance-1	1.00	0.001	1.000	Excellent
Stress tolerance-2	0.90	0.010	0.899	Excellent
Stress tolerance-3	1.00	0.001	1.000	Excellent
Stress tolerance-4	1.00	0.001	1.000	Excellent
Stress tolerance-5	1.00	0.001	1.000	Excellent
Stress tolerance-6	1.00	0.001	1.000	Excellent
Frustration tolerance-1	1.00	0.001	1.000	Excellent
Frustration tolerance-2	1.00	0.001	1.000	Excellent
Frustration tolerance-3	1.00	0.001	1.000	Excellent
Frustration tolerance-4	0.90	0.010	0.899	Excellent
Frustration tolerance-5	1.00	0.001	1.000	Excellent
Frustration tolerance-6	1.00	0.001	1.000	Excellent

I-CVI stands for Item Content Validity Index, *Pc* for probability of random consistency in experts’ ratings, and K^*^ for Corrected Content Validity Index.

#### Participants

This study adopted convenience cluster sampling to cover as many different types of work in special operations as possible so as to enhance the representativeness and universality of the results on psychological stability. Data were collected using the Wenjuanxing (online platform). The inclusion criterion for participants was current employment as special operations personnel. The exclusion criteria were: (1) presence of major physical disabilities, and (2) presence of mental diseases and psychological disorders. A total of 301 initial questionnaires were collected. After applying the exclusion criteria (e.g., incorrect answers to mandatory questions, selecting the same option repeatedly, and regular response patterns), 258 valid questionnaires remained, yielding an effective response rate of 85.71%. The demographic characteristics of the participants are presented in Table 6. Age ranged from 19 to 60 years (*M* = 42.74, SD = 10.40), and length of service ranged from 1 to 37 years (*M* = 13.53, SD = 9.64).

**Table 6:** Demographic information of participants (*N* = 258).

Characteristics	Category	Participants (n)	Proportion (%)
Gender	Male	226	87.60
	Female	32	12.40
Highest Education Level	Master’s Degree and Above	11	4.26
	Bachelor’s Degree	45	17.44
	Junior College Degree and Below	202	78.30
Only Child Status	Yes	70	27.13
	No	188	72.87
Marital Status	Unmarried	42	16.28
	Married	197	76.36
	Divorced	19	7.36
Job Type	Coal Mine Safety Operation	103	39.92
	Electrical Operation	77	29.84
	High-Altitude Operation	19	7.36
	Emergency Rescue Operation	19	7.36
	Confined Space Safety Operation	15	5.81
	Firecracker and Firework Safety Operation	11	4.26
	Other Special Operations	14	5.43

#### Statistical analysis

The 258 valid questionnaires of the initial PSS (with 39 items) were incorporated into the statistical analysis. Subsequent analyses—including item analysis, item-total correlation analysis, and EFA—were carried out via SPSS 29.0 software.

### Results

#### Item analysis and item-total correlation analysis

The total scores of the scale were sorted, assigning the top 27% of the sample to the high-score group and the bottom 27% to the low-score group (Liu et al., 2025). Differences in all items between the two groups were then analyzed using an independent samples *t*-test, and the results showed that all items had significant differences between the two groups (*p* < 0.001); therefore, 39 items were retained. In addition, correlation analysis of each item score with the total scale score indicated that one item had a correlation coefficient (*r*) of 0.39, below the statistical threshold of 0.40; thus, it was deleted. Finally, 38 items were retained.

#### EFA and model modification

The results of the Bartlett test of sphericity (*χ^2^* = 4872.517, df = 406, *p* < 0.001) and Kaiser–Meyer–Olkin test (KMO = 0.943) showed that the data were suitable for EFA. Principal component analysis (PCA) coupled with the maximum variance rotation method was employed to extract factors with eigenvalues exceeding 1. Factors and items that failed to meet the criteria were excluded according to the following thresholds: (1) factor loading < 0.50; (2) communality < 0.2; (3) the number of items in a common factor was less than 3; (4) items with cross-loading; (5) items with ambiguity or obviously improper classification. Each time an item was excluded, EFA was repeated until no items met the exclusion criteria (Liu et al., 2025). The initial scale ultimately excluded 9 items and retained 29 items. The variance of these 29 items was summarized into five common factors, with a cumulative contribution rate of 65.90%. Factor loadings for all items ranged from 0.511 to 0.830 (see Table 7).

**Table 7:** The five characteristic dimensions of the PSS.

Test items	SC	C	ER	DT	SFT	Commonality
I believe I can accomplish challenging tasks independently.	0.778					0.733
I can handle things independently at work.	0.759					0.711
Many people appreciate my talents or abilities.	0.736					0.632
I believe I fully possess the knowledge and skills required for my current job.	0.685					0.576
No matter what I do, I believe I can do it well.	0.670					0.615
Even without supervision, I still take my work tasks seriously.		0.830				0.808
I work hard and fulfill my duties with dedication.		0.827				0.805
I am serious and responsible in what I do.		0.797				0.806
I clearly understand my own responsibilities and obligations.		0.767				0.709
When mistakes occur at work, I never shirk responsibility.		0.732				0.679
I can handle things calmly and peacefully.			0.720			0.665
I am planned and organized in doing things.			0.677			0.619
I can control my temper well.			0.651			0.584
Before making a decision, I will first conduct a calm analysis.			0.626			0.663
I am not easily flustered when facing problems.			0.622			0.642
Before making a decision, I will fully consider all possible risks and consequences.			0.582			0.649
When facing complex problems, I can grasp the key points of the issue.			0.579			0.657
When I am focused on something, other things around me cannot easily distract me.				0.780		0.737
I am not easily affected by small things in life.				0.721		0.696
When I am concentrating on work, the surrounding noise cannot easily affect me.				0.645		0.522
After quarreling with my family, my work state will not be affected.				0.584		0.553
When expressing my own opinions, I am not easily led off topic by others’ words.				0.578		0.579
I believe that failure is the mother of success.					0.732	0.685
When I fail to get a promotion, I will still work hard.					0.673	0.678
When I am frequently criticized by leaders, I will still go all out to do my current job well.					0.671	0.701
I believe pressure is part of work and can help me grow better.					0.605	0.643
When in adversity, I will try every means to change the current situation.					0.561	0.652
When the goal is hindered, I will try to seek new methods to achieve it.					0.555	0.550
When facing high-intensity work tasks, I can maintain an optimistic attitude.					0.511	0.560
Rotated eigenvalue	3.634	3.957	4.024	3.876	3.619	
Rotated contribution rate	12.53%	13.64%	13.88%	13.37%	12.48%	65.90%
Internal consistency reliability	0.862	0.916	0.895	0.846	0.887	0.953

SC stands for self-confidence, C for conscientiousness, ER for equanimity and rationality, DT for disturbance tolerance, and SFT for stress and frustration tolerance.

Based on the results of EFA, five dimensions of the PSS with 29 items were developed. Three dimensions—self-confidence, conscientiousness, and disturbance tolerance—each contained 5 items. The two independent dimensions of equanimity and rationality were merged into a single dimension, which contained 7 items. Similarly, stress tolerance and frustration tolerance were merged into another dimension, also containing 7 items.

## Study 3: validation of the PSS for special operations personnel

In Study 3, the PSS developed in Study 2 was applied to a new sample. CFA was used to verify the five factors of the PSS. In this process, not only the discriminant validity, convergent validity, criterion-related validity and internal consistency reliability of the scale were evaluated, but the model fit indices were also tested to ensure the psychometric properties of the scale (Liu et al., 2025).

### Methods

#### Participants

A convenience cluster sampling method was employed to ensure the sample covered as many different types of special operations jobs as possible. Data were collected using the Wenjuanxing (online platform), and distributed by the responsible personnel of the special operations units. The inclusion criterion for participants was current employment as special operations personnel. The exclusion criteria were: (1) presence of major physical disabilities, and (2) presence of mental diseases and psychological disorders. A total of 766 initial questionnaires were collected. After applying exclusion criteria (e.g., incorrect answers to mandatory questions, selecting the same option repeatedly, and regular response patterns), 697 valid questionnaires were obtained, yielding an effective response rate of 90.99%. Demographic information of the participants is presented in Table 8. Participants’ age ranged from 18 to 59 years (*M* = 34.97, SD = 10.22), and their length of service ranged from 0.5 to 40 years (*M* = 10.31, SD = 9.14).

**Table 8:** Demographic information of participants (*N* = 697).

Characteristics	Category	Participants (n)	Proportion (%)
Gender	Male	621	89.10
	Female	76	10.90
Highest Education Level	Master’s Degree and Above	28	4.02
	Bachelor’s Degree	438	62.84
	Junior College Degree and Below	231	33.14
Only Child Status	Yes	309	44.33
	No	388	55.67
Marital Status	Unmarried	236	33.86
	Married	428	61.41
	Divorced	33	4.73
Job Type	Emergency Rescue Operation	449	64.42
	Coal Mine Safety Operation	102	14.63
	Electrical Operation	77	11.05
	High-Altitude Operation	25	3.59
	Confined Space Safety Operation	17	2.44
	Welding and Thermal Cutting Operation	9	1.29
	Other Special Operations	18	2.58

#### Measurements

The 29-item PSS developed in Study 2 was used as the measurement tool to be validated. To test the criterion-related validity, this study employed the GAD-7 (Spitzer et al., 2006; He et al., 2010), the PHQ-9 (Spitzer et al., 1999; Bian et al., 2009), the Positive Mental Health Scale (PMHS) (Lukat et al., 2016; Ding et al., 2023), and the 10-item Connor-Davidson Resilience Scale (CD-RISC-10) (Connor and Davidson, 2003; Wang et al., 2010) as criterion tools. The PSS uses a 5-point Likert scale (1–5; total score: 29–145). The GAD-7 consists of 7 items rated on a 4-point scale (0–3; total score: 0–21). The PHQ-9 has 9 items using a 4-point scale (0–3; total score: 0–27). The PMHS includes 9 items on a 4-point scale (1–4; total score: 9–36). The CD-RISC-10 contains 10 items on a 5-point scale (0–4; total score: 0–40). The GAD-7, PHQ-9, and PMHS have all demonstrated good internal consistency reliability in Chinese adult samples, with coefficients of 0.781 (Han et al., 2025), 0.854 (Tang et al., 2025), and 0.950 (Ding et al., 2023), respectively. In this study, the Cronbach’s *α* coefficients were 0.921 for the GAD-7, 0.908 for the PHQ-9, 0.952 for the PMHS, 0.946 for the CD-RISC-10, and 0.962 for the PSS. These values indicate acceptable internal consistency reliability for all questionnaires.

In addition, Harman’s single-factor test was used to test for common method bias in this sample. The results showed that the explanation rate of the first factor was 38.415%, which was lower than the critical standard of 40% (Podsakoff et al., 2003), indicating that there is no serious common method bias in the data of this study.

#### CFA

AMOS 27.0 was used to analyze the PSS developed in Study 2. According to the requirements of psychometric evaluation indicators commonly used in the field of questionnaire development, the selected fitness indicators include chi-square degrees of freedom ratio (*χ*^2^/df), root mean square error of approximation (RMSEA), standardized root mean square residual (SRMR), comparative fit index (CFI), Tucker–Lewis index (TLI), incremental fit index (IFI), and normed fit index (NFI).

### Results

#### CFA

**Figure 1:**
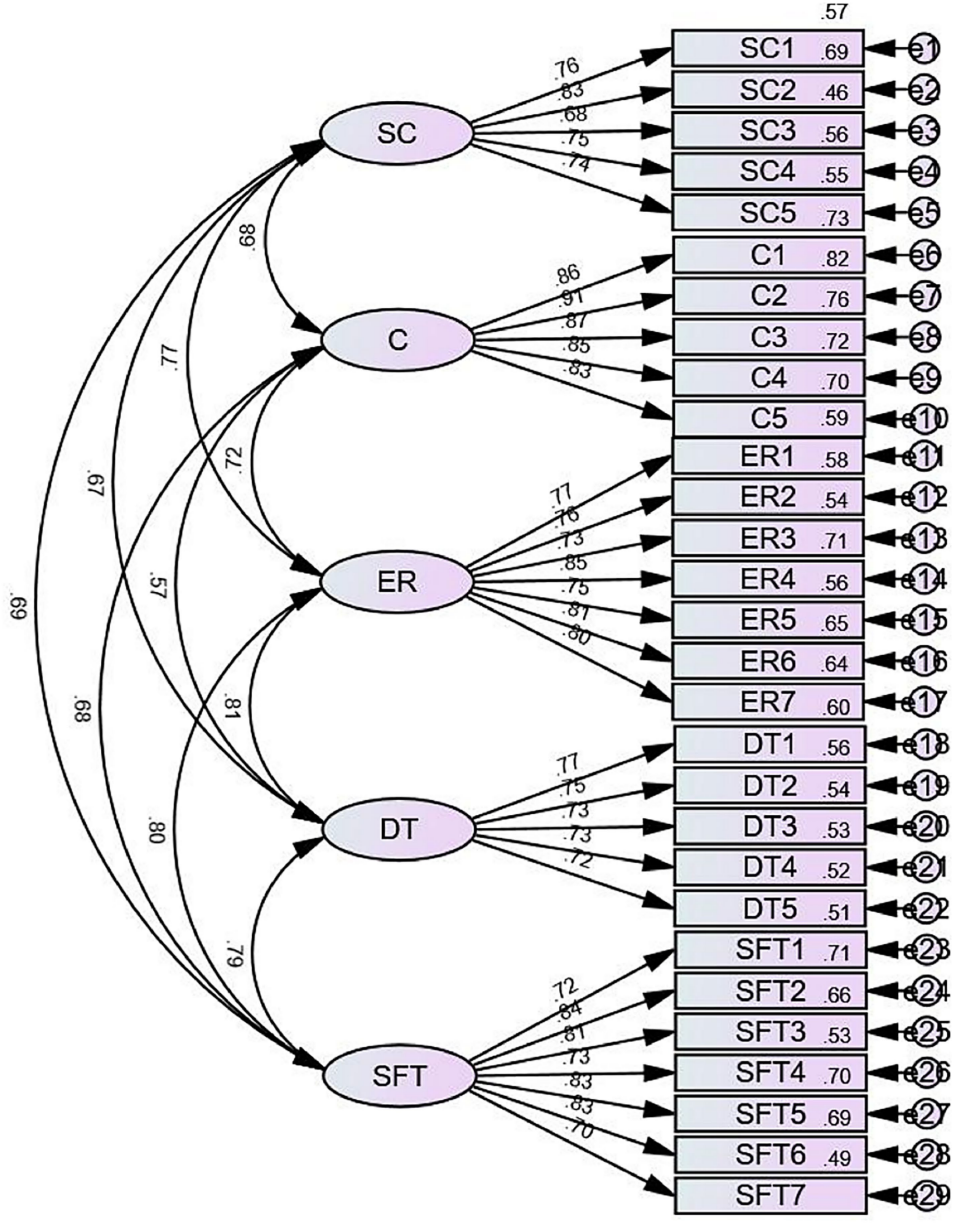
The results of CFA. SC stands for self-confidence, C stands for conscientiousness, ER stands for equanimity and rationality, DT stands for disturbance tolerance, SFT stands for stress and frustration tolerance.

To further verify the five-dimensional model of psychological stability, the study used AMOS 27.0 to conduct CFA (Collier, 2020). According to the common criteria of model fitting, a good model fit is indicated when *χ*^2^/df < 3, CFI > 0.90, TLI > 0.90, IFI > 0.90, NFI > 0.90, SRMR < 0.05, and RMSEA < 0.08. All fit indices of the five-dimensional model of psychological stability were demonstrated to be within the acceptable range (Table 9 and Figure 1). In addition, the standardized loading of each measurement item in the five-dimensional model on the corresponding factors ranged from 0.679 to 0.906, which could better explain the observed variables, indicating that the PSS developed in this study had good convergent validity.

**Table 9:** Model fit results of the PSS (*N* = 697).

Model	*χ* ^2^	*df*	*χ*^2^/*df*	CFI	TLI	IFI	NFI	SRMR	RMSEA
Five-factor model	1336.081^***^	367	3.641	0.934	0.927	0.935	0.912	0.026	0.062

***p < 0.001.

#### Discriminant validity

This study used the square root of the average variance extracted (AVE) to test the discriminant validity of the questionnaire. If the square root of the AVE for a given factor is greater than the absolute values of its correlation coefficients with all other factors, and this condition holds for all factors, then the scale is demonstrated to have good discriminant validity (Fornell and Larcker, 1981). The results are presented in Table 10, where the diagonal values represent the square roots of the AVE and the other values represent the Spearman correlation coefficients. All dimensions showed significant correlations (*p* < 0.01), and all correlation coefficients were smaller than the square roots of their respective AVE, indicating that the PSS demonstrates good discriminant validity.

**Table 10:** Discriminant validity analysis results of the PSS (*N* = 697).

Dimensions	Self-confidence	Conscientiousness	Equanimity and rationality	Disturbance tolerance	Stress and frustration tolerance
Self-confidence	0.753				
Conscientiousness	0.590^**^	0.863			
Equanimity and rationality	0.655^**^	0.651^**^	0.782		
Disturbance tolerance	0.561^**^	0.525^**^	0.708^**^	0.741	
Stress and frustration tolerance	0.604^**^	0.525^**^	0.736^**^	0.708^**^	0.783

**p < 0.01.

#### Convergent validity

The study used AVE and Composite Reliability (CR) to assess the convergent validity of the questionnaire. An AVE value greater than 0.5 indicates good convergent validity (Fornell and Larcker, 1981). The results, presented in Table 11, show that all dimensions had AVE values greater than 0.5 and CR values greater than 0.8, indicating that the scale demonstrates good convergent validity.

**Table 11:** Convergent validity analysis results of the PSS (*N* = 697).

Dimensions	AVE	CR
Self-confidence	0.566	0.867
Conscientiousness	0.745	0.936
Equanimity and rationality	0.612	0.917
Disturbance tolerance	0.549	0.859
Stress and frustration tolerance	0.613	0.917

#### Criterion-related validity

The criterion-related validity was evaluated by the correlations between the scores of the PSS and those of the GAD-7, the PHQ-9, the PMHS, and the CD-RISC-10. The results indicated that the total score and scores on each dimension of the PSS were negatively correlated with those of the GAD-7 and PHQ-9, while positively correlated with those of the PMHS and CD-RISC-10. Results showed that the questionnaire has good criterion-related validity (Table 12).

**Table 12:** Correlation between the PSS and the criterion scales (*N* = 697).

Criterion scales	Total score of psychological stability	Self-confidence	Conscientiousness	Equanimity and rationality	Disturbance tolerance	Stress and frustration tolerance
GAD-7	−0.490^**^	−0.368^**^	−0.339^**^	−0.428^**^	−0.461^**^	−0.454^**^
PHQ-9	−0.487^**^	−0.389^**^	−0.336^**^	−0.417^**^	−0.443^**^	−0.450^**^
PMHS-9	0.625^**^	0.487^**^	0.464^**^	0.551^**^	0.529^**^	0.603^**^
RISC-10	0.618^**^	0.502^**^	0.452^**^	0.526^**^	0.527^**^	0.592^**^

**p < 0.01.

#### Internal reliability

The Cronbach’s *α* coefficients for the total score of the PSS and its respective dimensions — self-confidence, conscientiousness, equanimity and rationality, disturbance tolerance, and stress and frustration tolerance — are 0.962, 0.863, 0.935, 0.914, 0.855, and 0.912, respectively. The Guttman split-half reliability coefficient is 0.893. All values exceeded the conventional threshold of 0.70, demonstrating good internal consistency reliability (Collier, 2020).

## Discussion

This study employed both qualitative and quantitative research methods to construct a theoretical model of psychological stability for special operations personnel and develop an assessment tool. Study 1 adopted qualitative research based on grounded theory and the Delphi method, and constructed a 7-dimensional theoretical model of psychological stability. The seven dimensions included self-confidence, conscientiousness, equanimity, rationality, stress tolerance, disturbance tolerance, and frustration tolerance. Based on the findings of Study 1, Study 2 developed an item pool for the PSS, and developed a 29-item PSS through content validity analysis, item analysis, item-total correlation analysis, and EFA. Meanwhile, the original seven dimensions of psychological stability were merged into five dimensions. Equanimity and rationality were combined into one dimension, as were stress tolerance and frustration tolerance. Study 3 conducted CFA and reliability-validity testing across samples to ensure the validity and reliability of this scale.

### Conceptual structure of the psychological stability in special operations personnel

This study employed a grounded theory approach to develop a conceptual framework for psychological stability. According to Homeostasis and Adaptation Level Theory, psychological stability is defined as an individual’s quality and ability to effectively regulate and control their mental processes when facing stimuli, thereby maintaining normal functional performance and psychological well-being under stressful or challenging conditions. It is characterized by adaptability and developmental potential. Through quantitative research, we identified five dimensions of psychological stability: self-confidence, conscientiousness, and equanimity and rationality belong to psychological qualities, while disturbance tolerance and stress and frustration tolerance belong to psychological capabilities. Under mild to moderate stress conditions, individuals mainly rely on psychological qualities to regulate and maintain psychological stability. If the stress intensity increases or persists, individuals achieve a certain degree of adaptation and establish a new stable state by synergistically employing psychological qualities and psychological capabilities. However, if the stress is too intense or prolonged and exceeds the capacity of psychological regulation and control, it may lead to decision-making errors, emotional dyscontrol, behavioral deviations, and even psychological disorders.

This study employed the grounded theory approach to construct a conceptual framework for psychological stability. Allostasis theory posits that when individuals face changes in internal and external environments, they ultimately achieve and maintain a new state of adaptive stability compatible with the environment through the regulation of physiology, psychology, and behavior—building on their original state of stability (Gao and Yu, 2020). Guided by the philosophy of mind–body unity, we believe that psychological stability is equally important as physiological homeostasis, and they together form an individual’s overall stability. Furthermore, we contend that psychological stability, similar to physiological homeostasis, has a certain homeostatic foundation. From the perspective of personality psychology, self-confidence, conscientiousness, and equanimity and rationality are likely to be the homeostatic foundations of psychological stability. This is because self-confidence, as self-reported through questionnaires, is more akin to a personality trait (Burns et al., 2016). We believe that self-confidence serves as the driving force behind psychological stability, without which psychological stability would not be possible. As a personality trait, conscientiousness is an effective predictor of job performance (Halim et al., 2011) and a strong indicator of career success (Moldzio et al., 2024). We argue that conscientiousness exerts a constraining effect on maintaining psychological stability: individuals with a stronger conscientiousness are more likely to abide by rules, thereby becoming more reliable and demonstrating better psychological stability. Additionally, Ajose suggests that emotional stability can facilitate rational decision-making and effective communication (Ajose, 2025). Emotional stability is a key determinant of psychological stability, and psychological stability, in turn, can mitigate cognitive biases (Ady, 2018). Although in Study 1, we classified equanimity and rationality as two distinct dimensions, they are mutually reinforcing. Based on the results of Study 2 and Study 3, we ultimately merged equanimity and rationality.

**Figure 2:**
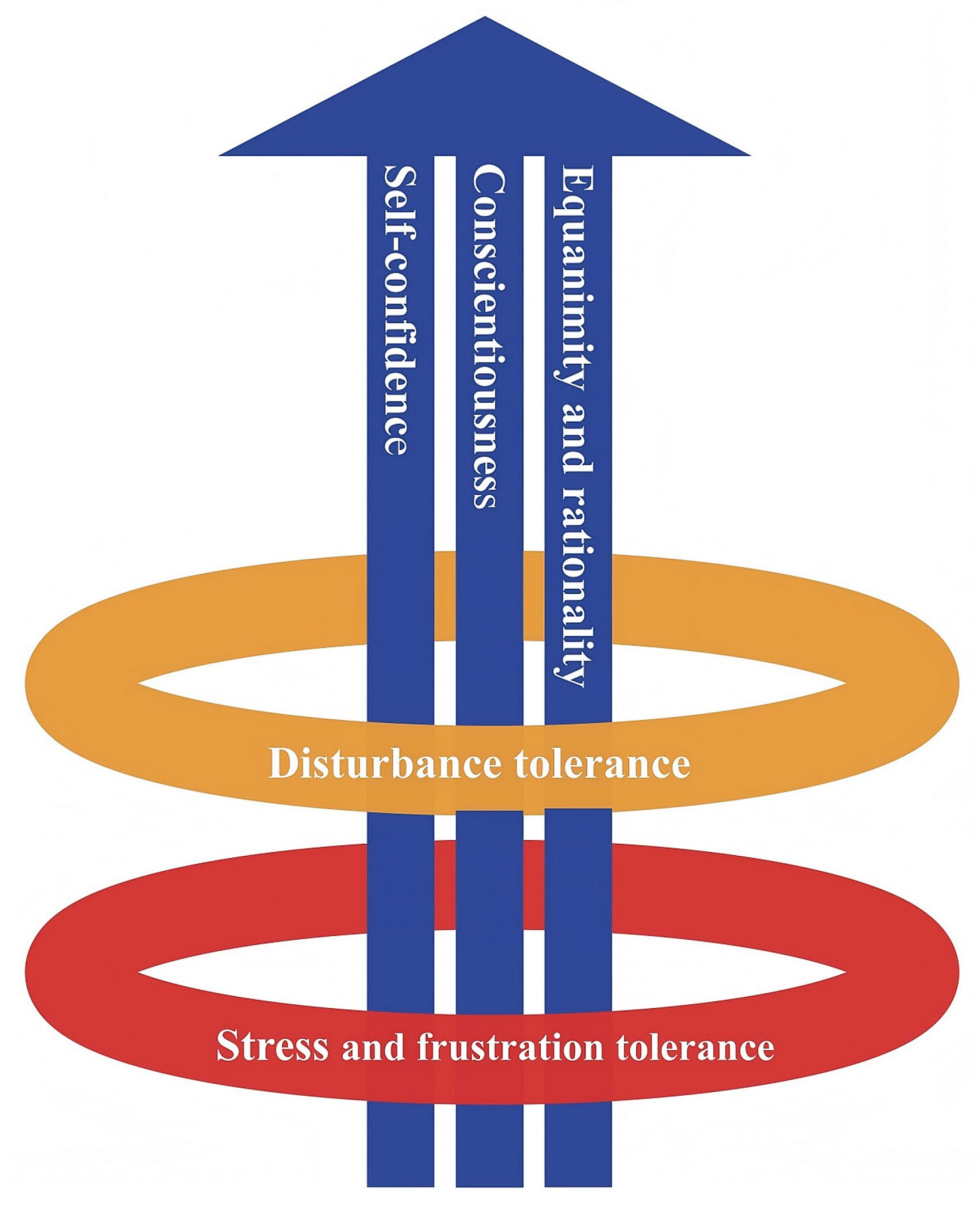
Five-factor model of psychological stability.

The Adaptation Level Theory emphasizes that when individuals experience maladaptation to new external stimuli, their psychological systems respond to deviations from the optimal adaptation level (Duan et al., 2020). Both the theory of Allostasis and the Adaptation Level Theory highlight the role of adaptation, indicating that adaptation to external stimuli is another crucial aspect of psychological stability. From the perspective of developmental psychology, disturbance tolerance and stress and frustration tolerance are likely to serve as the key adaptive systems for psychological stability. For military personnel, maintaining attention stability and flexibility is essential in high-pressure operational environments (Mecheri, 2025). High-intensity stress can threaten the psychological stability of special police, impairing their ability to perform tasks safely (Jäger et al., 2023). We propose that attention stability and flexibility are the core of disturbance tolerance. Specifically, this dimension also includes resistance to temptations, noise, and minor incidents, which are not typically categorized as stress. Furthermore, in Study 1, we distinguished between stress tolerance and frustration tolerance as two separate dimensions. However, the results of Study 2 and Study 3 supported the merging of these two dimensions into one. As a result, a five-dimensional model of psychological stability for special operations personnel was ultimately established (see Figure 2).

### Reliability and validity of the PSS

Previous studies on tools for assessing psychological stability have largely focused on mental health, psychological resilience, and psychological safety, but these tools have often failed to directly and comprehensively capture the characteristics of psychological stability. The PSS developed in this study addresses this shortcoming by dividing the concept of psychological stability into five dimensions: self-confidence, conscientiousness, equanimity and rationality, disturbance tolerance, and stress and frustration tolerance. During the validation process, the scale demonstrated strong reliability and validity, with each dimension exhibiting good internal consistency. This suggests that each dimension may also serve as a valid sub-scale for application in other studies. Additionally, the PSS showed a significant negative correlation with the GAD-7 and the PHQ-9, and a significant positive correlation with the PMHS and the CD-RISC-10. This indicates that future research should further explore the causal relationship between psychological stability and mental health. Finally, before administering the scale, we had already conducted expert consultations on the correlation between the scale items and dimensions. Although the scale dimensions were merged in Study 2, this change did not impact the correlation between items and dimensions; therefore, no additional expert consultation was conducted in Study 3 to reassess the content validity.

### Limitations and future research directions

Although the present study employed a mixed-methods approach, it had several limitations. First, the research sample was exclusively recruited from China, which may restrict the generalizability of the findings to different cultural contexts in other countries. Second, the study utilized a cross-sectional design, only capturing psychological stability in special operations personnel at a single time point. To explore how psychological stability evolves over time in this group, longitudinal studies are essential. Lastly, despite the fact that the PSS developed in this study demonstrated good reliability and validity among special operations personnel, its applicability to other high-risk and high-pressure occupational populations remains to be further validated. Future research may proceed in two directions: one is to adopt a longitudinal design to uncover the underlying mechanisms of psychological stability, and the other is to evaluate the applicability of the PSS across different countries and diverse high-risk, high-pressure occupational groups.

## Conclusion

This study adopted both qualitative and quantitative research methods, identified the dimensions of psychological stability among special operations personnel, and developed a 29-item PSS with good reliability and validity. Thus, it provides an important basis for the assessment and management of psychological stability in special operations personnel.

## Data Availability

The datasets presented in this study can be found in online repositories. The names of the repository/repositories and accession number(s) can be found: https://doi.org/10.57760/sciencedb.37200.
