# Supplementary material for: Development and validation of the psychological stability scale for special operations personnel in China
Source: Front Psychol. 2026 Feb 26;17:1731159. doi: 10.3389/fpsyg.2026.1731159 (PMC12979458; doi:10.3389/fpsyg.2026.1731159)
Supplement: Supplementary file 1 [file Table_1.docx]

Supplementary Material

**Psychological Stability Scale for Special Operations Personnel**

| **Test Items** | **Strongly Disagree** | **Disagree** | **Neutral** | **Agree** | **Strongly Agree** |
| --- | --- | --- | --- | --- | --- |
| 1. I believe I can accomplish challenging tasks independently. | 1 | 2 | 3 | 4 | 5 |
| 1. I can handle things independently at work. | 1 | 2 | 3 | 4 | 5 |
| 1. Many people appreciate my talents or abilities. | 1 | 2 | 3 | 4 | 5 |
| 1. I believe I fully possess the knowledge and skills required for my current job. | 1 | 2 | 3 | 4 | 5 |
| 1. No matter what I do, I believe I can do it well. | 1 | 2 | 3 | 4 | 5 |
| 1. Even without supervision, I still take my work tasks seriously. | 1 | 2 | 3 | 4 | 5 |
| 1. I work hard and fulfill my duties with dedication. | 1 | 2 | 3 | 4 | 5 |
| 1. I am serious and responsible in what I do. | 1 | 2 | 3 | 4 | 5 |
| 1. I clearly understand my own responsibilities and obligations. | 1 | 2 | 3 | 4 | 5 |
| 1. When mistakes occur at work, I never shirk responsibility. | 1 | 2 | 3 | 4 | 5 |
| 1. I can handle things calmly and peacefully. | 1 | 2 | 3 | 4 | 5 |
| 1. I am planned and organized in doing things. | 1 | 2 | 3 | 4 | 5 |
| 1. I can control my temper well. | 1 | 2 | 3 | 4 | 5 |
| 1. Before making a decision, I will first conduct a calm analysis. | 1 | 2 | 3 | 4 | 5 |
| 1. I am not easily flustered when facing problems. | 1 | 2 | 3 | 4 | 5 |
| 1. Before making a decision, I will fully consider all possible risks and consequences. | 1 | 2 | 3 | 4 | 5 |
| 1. When facing complex problems, I can grasp the key points of the issue. | 1 | 2 | 3 | 4 | 5 |
| 1. When I am focused on something, other things around me cannot easily distract me. | 1 | 2 | 3 | 4 | 5 |
| 1. I am not easily affected by small things in life. | 1 | 2 | 3 | 4 | 5 |
| 1. When I am concentrating on work, the surrounding noise cannot easily affect me. | 1 | 2 | 3 | 4 | 5 |
| 1. After quarreling with my family, my work state will not be affected. | 1 | 2 | 3 | 4 | 5 |
| 1. When expressing my own opinions, I am not easily led off topic by others' words. | 1 | 2 | 3 | 4 | 5 |
| 1. I believe that failure is the mother of success. | 1 | 2 | 3 | 4 | 5 |
| 1. When I fail to get a promotion, I will still work hard. | 1 | 2 | 3 | 4 | 5 |
| 1. When I am frequently criticized by leaders, I will still go all out to do my current job well. | 1 | 2 | 3 | 4 | 5 |
| 1. I believe pressure is part of work and can help me grow better. | 1 | 2 | 3 | 4 | 5 |
| 1. When in adversity, I will try every means to change the current situation. | 1 | 2 | 3 | 4 | 5 |
| 1. When the goal is hindered, I will try to seek new methods to achieve it. | 1 | 2 | 3 | 4 | 5 |
| 1. When facing high-intensity work tasks, I can maintain an optimistic attitude. | 1 | 2 | 3 | 4 | 5 |

To assess the psychological stability of special operations personnel (high-risk, high-pressure workers), we have specifically designed this scale titled 'Psychological Stability of Special Operations Personnel'. There is no right or wrong in the scale, please answer based on your true feelings.

**Interview Outline on Psychological Stability of Special Operations Personnel**

Dear Research Participant (Respondent):

Hello! I am a researcher from the Army Medical University. At present, we are undertaking a research project focused on psychological stability. The information you provide will be highly valuable to our work, and we would greatly appreciate your careful and honest responses. The results of this interview will only be used for academic research, and your personal identity information will be strictly confidential. We invite you to share your insights into psychological stability within special working environments—from the perspective of special operations personnel—drawing on your personal experiences and professional perceptions. This discussion will primarily cover the following key questions:

1. Have you heard of psychological stability?
2. What do you think psychological stability refers to?
3. What do you think psychological stability consists of ? Please give some examples to illustrate.
4. Do you consider yourself a person with high or low psychological stability? And why do you think so?
5. What events in your life affect your psychological stability? Please provide examples if possible.
6. What events at work affect your psychological stability? Could you please give some examples?
7. Is there anything else you'd like to add regarding psychological stability?

**Expert Consultation on the Dimensions and Connotations of Psychological Stability for Special Operations Personnel (The First-Round)**

Dear Experts:

Hello! In the preliminary stage, we developed the conceptual dimensions and connotations of psychological stability in special operations personnel through in-depth interviews and grounded theory.

Please carefully review the following dimensions and connotations of psychological stability, and mark a "√" in the importance column (very important, relatively important, general, less important, unimportant) for each dimension. If there are any inappropriate parts in the dimension names or connotation descriptions, or if any dimensions need to be added or deleted, please provide your revision suggestions.

| Dimensions | The connotation of dimensions | The importance of dimensions | | | | |
| --- | --- | --- | --- | --- | --- | --- |
|  |  | Very Important | Relatively Important | General | Less Important | Unimportant |
| Self-confidence | The degree of an individual's trust and affirmation in their own abilities, value, and competence in various tasks. |  |  |  |  |  |
| Conscientiousness | The degree to which an individual consciously fulfills their obligations and proactively takes responsibility for the consequences of their actions. |  |  |  |  |  |
| Emotional regulation | An individual's selection and use of emotional regulation strategies (such as expression suppression, cognitive reappraisal, attention allocation, etc.) to successfully regulate their own emotions. |  |  |  |  |  |
| Decision  making | An individual's comprehensive consideration of various factors, objective analysis, reasonable judgment, and selection of the optimal solution when facing problems. |  |  |  |  |  |
| Disturbance Tolerance | The ability of individuals to maintain concentrated attention, clear thinking, and normal psychological activities and behavior. |  |  |  |  |  |
| Stress Tolerance | The ability of individuals to maintain normal psychological and physiological functions when subjected to external stressors. |  |  |  |  |  |
| Frustration Tolerance | The ability of individuals to restore psychological balance, adjust behavioral strategies, and positively cope when encountering frustration situations (such as failure, loss, obstacles, etc.). |  |  |  |  |  |
| Expert revision suggestions on dimension names and connotations： | | | | | | |

**Expert Consultation on the Conceptual Dimensions and Connotations of Psychological Stability in Special Operations Personnel (The Second-Round)**

Dear Experts:

Hello! Thank you sincerely for the valuable feedback you provided on the dimensions and connotations of psychological stability for special operations personnel during the first round of consultation. After synthesizing expert opinions, reviewing the raw interview data, and consulting relevant literature, we have made corresponding revisions to the descriptions of the dimensions and connotations. The purpose of this second-round consultation is to invite you to share your valuable feedback once more on the revised descriptions of the dimensions and connotations.

Please mark a "√" in the importance column (very important, relatively important, general, less important, unimportant) for each dimension. Additionally, if you find any inappropriate parts in the dimension names or connotation descriptions, or if there are dimensions that need to be added or deleted, please feel free to provide your revision suggestions.

| Dimensions | The connotation of dimensions | The importance of dimensions | | | | |
| --- | --- | --- | --- | --- | --- | --- |
|  |  | Very Important | Relatively Important | General | Less Important | Unimportant |
| Self-confidence | The trait of individuals who believe in and affirm their own abilities. |  |  |  |  |  |
| Conscientiousness | The trait of individuals who consciously fulfill their obligations, actively take responsibility for the consequences of their own behavior, and have the courage to undertake the mission. |  |  |  |  |  |
| Equanimity | The trait of individuals who can maintain emotional stability, peace and calm through emotional regulation strategies |  |  |  |  |  |
| Rationality | The trait of individuals who can make wise decisions through comprehensive consideration, objective analysis, and reasonable judgment |  |  |  |  |  |
| Disturbance Tolerance | The ability of individuals to effectively regulate attention stability and flexibility in the face of external noise, temptation and other disturbances in the process of achieving goals. |  |  |  |  |  |
| Stress Tolerance | The ability of individuals to maintain the normal functioning of their psychological and physiological functions when they believe that their own abilities may be difficult to meet external demands. |  |  |  |  |  |
| Frustration Tolerance | The ability of individuals to quickly restore their psychological balance and actively respond to the realistic failure when their goals are blocked or expectations fall short. |  |  |  |  |  |
| Expert revision suggestions on dimension names and connotations： | | | | | | |
